# A cross section through mosquitoes of Bosnia and Herzegovina: Barcodes, blood meals and pathogens

**DOI:** 10.1016/j.onehlt.2025.101246

**Published:** 2025-10-15

**Authors:** Ina Hoxha, Jovana Dervović, Maria Sophia Unterköfler, Lisa Schlamadinger, Tanto Situmorang, Hans-Peter Fuehrer, Adelheid G. Obwaller, Karin Sekulin, Jeremy V. Camp, Josef Harl, Julia Walochnik, Amer Alić, Edwin Kniha

**Affiliations:** a Center for Pathophysiology, Infectiology and Immunology, Institute of Specific Prophylaxis and Tropical Medicine, Medical University Vienna, Vienna, Austria; b Department of Clinical Sciences of Veterinary Medicine, Faculty of Veterinary Medicine, University of Sarajevo, Sarajevo, Bosnia and Herzegovina; c Department of Biological Sciences and Pathobiology, University of Veterinary Medicine Vienna, Vienna, Austria; d Division of Science, Research and Development, Federal Ministry of Defence, Vienna, Austria; e Armaments and Defence Technology Agency, Vienna, Austria; f Center for Virology, Medical University Vienna, Vienna, Austria; g Institute of Pathology, Department of Pathobiology, University of Veterinary Medicine Vienna, Vienna, Austria

**Keywords:** *Aedes albopictus*, *Aedes japonicus*, Barcoding, *Culex*, *Dirofilaria*, Haplotyping, One health, West Nile virus

## Abstract

Mosquitoes are important vectors of human and animal pathogens, yet data on their diversity and vector potential remain scarce for the central Balkan country Bosnia and Herzegovina. This study aimed to assess mosquito species composition, associated pathogens, and the potential public health risks in BIH. Adult mosquitoes were collected with light traps, identified morphologically and by barcoding, and screened molecularly for various pathogens, including West Nile virus (WNV), *Dirofilaria* spp., *Trypanosoma* spp., and *Plasmodium* spp.

A total of 2691 mosquitoes of 17 species were identified, with *Culex pipiens*/*torrentium* being most abundant and new records of *Aedes albopictus* and *Ae. japonicus japonicus*. The first detection of WNV (lineage 2) RNA in mosquitoes in BIH highlights the potential risk of circulation in the region, aligning with findings from neighboring countries. In addition, DNA of filarioid nematodes (*Dirofilaria immitis*, *D. repens,* and *Setaria tundra*) were detected, underscoring their role as potential vectors of zoonotic dirofilariasis. Also, *Trypanosoma* and *Plasmodium* DNA was detected, warranting further investigation into the possible involvement of mosquitoes in their transmission.

The detection of invasive *Aedes* species and mosquito-borne pathogens emphasize the need for strengthened vector surveillance in southeastern Europe, particularly in BIH. This study provides the first barcode inventory of 17 mosquito species and novel molecular evidence of mosquito-borne pathogens in BIH, offering valuable baseline data for future epidemiological assessments and sustained entomological surveillance.

## 1 Introduction

Mosquitoes (Diptera: Culicidae) are important vectors of numerous disease-causing viruses and parasites world-wide, such as Dengue virus, West Nile virus, malaria-causing protozoa of the genus *Plasmodium*, or filarioid nematodes of the genus *Dirofilaria* [1]. In Europe, various endemic species of the genera *Anopheles*, *Aedes*, and *Culex* are of medical and veterinary importance as reservoirs and vectors of zoonotic pathogens to humans [2].

The recent introductions of *Aedes* (*Stegomyia*) *albopictus* (Skuse, 1894) and *Aedes* (*Hulecoeteomyia*) *japonicus japonicus* (Theobald, 1901) to Europe add two important vector species to the local fauna. Populations of both species have established successfully across Europe in the last decades, facilitated by human activity and the species' ability to adapt to a range of habitats, also in temperate regions [3,4].

The Balkans have a diverse mosquito fauna and are an important gateway of potential vector species to Europe; however, data are often fragmented or even lacking across countries, particularly in the central Balkan country Bosnia and Herzegovina (BIH) [5]. While data from neighboring countries such as Croatia [6] or the Republic of Serbia [7] highlight a rich mosquito fauna in the region, data on the endemic fauna in BIH are scarce. Recent VectorNet surveillance data provide valuable information on newly introduced non-endemic species and have confirmed the presence of *Ae. albopictus* in BIH in the last decade [8]. The first introduction events of *Ae. japonicus* date back to 2017 in Posavina region, close to the border with Croatia. More recent sampling efforts reported the establishment of *Ae. japonicus* in multiple additional regions [9].

The presence of *Ae. albopictus* and *Ae. japonicus* in BIH, along with their established populations across the Balkans, significantly heightens the risk of autochthonous circulation and transmission of arboviruses, such as Dengue virus (genus *Orthoflavivirus*) or Chikungunya virus (genus *Alphavirus*) [9,10]. Due to their laboratory vector competence for at least 20 arboviruses, these two species pose a major public health threat, requiring surveillance and vector control measures to mitigate potential outbreaks.

Among the arboviruses endemic to Europe, West Nile virus (“WNV”, *Flaviviridae*), a member of the Japanese encephalitis serogroup, is geographically the most widespread arbovirus [11]. The virus is maintained by mosquito species of the genus *Culex* and infects viremic wild birds [12,13]. Seasonal amplification of the virus via the enzootic transmission cycle between bird hosts and mosquito vectors may lead to spillover to incidental, dead-end hosts, including humans and horses, via infected mosquitoes [11,14]. In humans, WNV is asymptomatic but may cause an unspecific febrile illness in up to 20 % of infected individuals, and in rare cases West Nile neuroinvasive disease (WNND) may develop [15,16].

In the Balkans, WNV outbreaks have been documented in humans and animals in Albania and the Republic of Kosovo [17], BIH [18], Croatia [19], and the Republic of Serbia [20]. Despite the confirmed endemic circulation of WNV in neighboring regions, it has not been detected in mosquitoes in BIH.

In Europe, the most important zoonotic mosquito-borne nematodes are *Dirofilaria repens* and *Dirofilaria immitis* (Spirurida: Onchocercidae), being parasites of dogs, other domestic or wild carnivores, and occasionally humans [21]. *Dirofilaria immitis* has an almost worldwide distribution, whereas *D. repens* is only endemic in the Old World. The latter occurs in subcutaneous tissues, causing subcutaneous dirofilariosis [22], while *D. immitis* can cause severe conditions known as feline or canine heartworm disease [21]. *Aedes vexans*, *Ae. albopictus* and *Cx. pipiens* s.l. are considered the natural vectors of these nematodes, but many other species are also assumed to transmit them [23,24]. In addition, *Setaria tundra* is a filarioid nematode of cervids with zoonotic potential, causing peritonitis or neurological disorders in humans [[25], [26], [27]].

Various studies have confirmed the presence of *D. repens* and *D. immitis* in both dogs and mosquito vectors in the region [[28], [29], [30]], highlighting their role in maintaining the transmission cycle. Furthermore, canine dirofilariosis was investigated in dogs in BIH, revealing the presence of both *D. repens* (1.91 %) and *D. immitis* (3.11 %). The vast majority of sampled dogs had no travel history, suggesting that these infections were autochthonous [31]. Despite the zoonotic significance of *Dirofilaria* spp., detection and infection rates in mosquitoes have not been previously investigated in BIH.

To date, the distribution, diversity and ecology of mosquitoes of BIH and their vector status remain poorly documented. To address some of these gaps, our study aimed to provide a cross-section through the local mosquito fauna of central and north-eastern parts of BIH, analysing mosquito distribution including a *COI* barcode inventory, blood meal analysis as well as RNA/DNA-based pathogen screening.

## 2 Material & methods

### 2.1 Study area

**Fig. 1:**
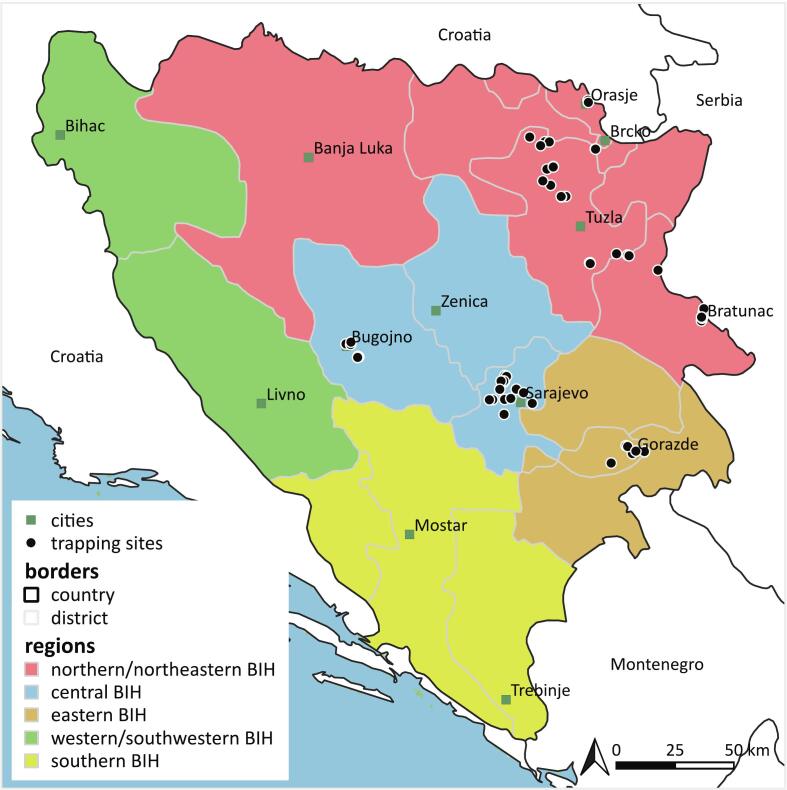
Mosquito trapping sites in Bosnia and Herzegovina. Data on the country borders and administrative divisions were obtained from Natural Earth (naturalearthdata.com).

The present study was conducted in Bosnia and Herzegovina, a country located west of the Balkan Peninsula in South-Eastern Europe (Fig. 1). BIH has a moderate climate with Mediterranean influences in the southern and mountainous part of the country. The central and eastern mountainous regions, along with the northeastern flatlands display a moderate climate with cold snowy winters and hot summers.

### 2.2 Mosquito sampling and morphological identification

Entomological surveys were conducted during 2022 and 2023 from July to September and from July to August, respectively. Up to four CDC miniature light traps (John W. Hock. Company, Gainesville, FL, USA) were used in each of the 56 locations (Fig. 1), with a total of 196 trap-nights [32]. Human landing catches were taken opportunistically as they may represent day-active species not frequently sampled in light traps. The selected regions comprised three of five geographical regions, namely central BIH, eastern BIH, and northern/northeastern BIH, including mostly private households as well as small- and large-scale animal farms (Fig. 1). Verbal consent was obtained from all homeowners prior to data collection, and records were consistently kept on coordinates, type of locality, trap placement (indoors or outdoors), and the presence of domestic animals and livestock.

Trapped specimens were identified morphologically based on identification keys of Becker et al. [33], sorted and pooled by species, trapping location and date and stored at −80 °C until further analyses.

### 2.3 Nucleic acid extraction

Pooled specimens (660 pools, maximum 23 specimens per pool) were homogenized in 0.5 mL Dulbecco's Modified Eagle Medium supplemented with 1 % penicillin/streptomycin, 10 μg/mL gentamicin, 20 % bovine serum albumin, and 0.25 μg/mL amphotericin B (both from Gibco, Thermo Fischer Scientific, Waltham, MA, USA). Two 3-mm diameter metal beads were added to each pool and the sample was homogenized with a TissueLyser bead mill (QIAGEN GmbH, Hilden, Germany) for 2 min of shaking at 30 Hz. The homogenate was subsequently cleared via centrifugation for 5 min at 14,000 rpm in a 4 °C benchtop centrifuge. Total nucleic acids were extracted from 200 μL supernatant per pool using the innuPREP AniPath DNA/RNA kit (iST Innuscreen, Berlin, Germany) on the KingFisher™ Apex Purification System.

### 2.4 DNA COI barcoding

DNA was isolated from single mosquitoes using the QIAamp® DNA Mini Kit 250 (QIAGEN, Hilden, Germany). For species-level identification, a barcoding PCR was conducted targeting a 658 base pair (bp) fragment of the cytochrome *c* oxidase subunit I (*COI*) gene, employing the primers LCO1490/HCO2198 following Folmer et al. [34]. Species of the *Anopheles maculipennis* complex were discriminated by ITS2 PCR using the primers 5.8SF/28SR developed by Collins and Paskewitz [35].

### 2.5 Blood meal analysis and DNA-based pathogen detection

Blood meal analysis was carried out using two primer sets: (1) 16S rDNA fragment with the primers L2513/H2714, following the protocol described by Kitano et al. [36], and (2) an avian-specific PCR targeting a 220-bp fragment of the cytochrome *b* (*Cytb*) gene using the primers L15330AV (L0)/H15551AV (H1) as described in Lee et al. [37].

All pools of samples were additionally tested by PCR for the presence of DNA of Filarioidea, *Plasmodium* spp., and *Trypanosoma* spp. (Table 1).

**Table 1:** Protocols of DNA-based pathogen detection by PCR applied in this study.

Organism Target (length)	Primer 5′-3′	Protocol	Reference
**Filarioidea**^a^ *COI* (668 bp)	COlint_F: TGATTGGTGGTTTTGGTAA COlint_R: ATAAGTACGAGTATCAATATC	94 °C/2 min; 8 cycles with 0.5 °C reduction/step: 94 °C/45 s, 51 °C/45 s, 72 °C/1.5 min; 25 cycles: 94 °C/45 s, 45 °C/45 s, 72 °C/1.5 min; 72 °C 7 min	[38]
***Plasmodium* spp.**^b^*Cytb* (∼617 bp)	Haem_NF1: CATATATTAAGAGAA**5**TATGGAG Haem NR3: ATAGAAAGATAAGAAATACCATTC	95 °C/2 min; 35 cycles: 95 °C/45 s, 50 °C/45 s, 72 °C/1 min; 72 °C 5 min	[39]
*Cytb* (∼480 bp)	Haem_F: ATGGTGCTTTCGATATATGCATG Haem_R2: GCATTATCTGGATGTGATAATGGT	95 °C/2 min; 35 cycles: 95 °C/45 s, 50 °C/45 s, 72 °C/1 min; 72 °C 5 min	
**Trypano-somatidae**^b^ 18S rRNA (∼1320 bp)	Tryp_18S_F1: GTGGACTGCCATGGCGTTGA Tryp_18S_R1: CAGCTTGGATCTCGTCCGTTGA	96 °C/5 min; 35 cycles: 94 °C/1 min, 56 °C/1 min, 72 °C/1 min; 72 °C 5 min	[40]
18S rRNA (∼960 bp)	Tryp_18S_F2: CGATGAGGCAGCGAAAAGAAATAGAG Tryp_18S_R2: GACTGTAACCTCAAAGCTTTCGCG	96 °C/5 min; 35 cycles: 94 °C/1 min, 56 °C/1 min, 72 °C/1 min; 72 °C 5 min	

a Touchdown PCR.

b Nested PCR.

All PCRs were performed in a final volume of 25 μL, using a 2 × EmeraldAmp® GT PCR Master Mix (Takara Bio Europe AB, Goteborg, Sweden) and an Eppendorf Mastercycler (Eppendorf AG, Hamburg, Germany). PCR products were visualized using a Gel Doc™ XR+ Imager (Bio-Rad Laboratories Inc., Hercules, CA, USA), and amplified fragments were purified with an Illustra™ GFX™ PCR DNA and Gel Purification Kit (GE Healthcare, Buckinghamshire, UK). The purified samples were sent to Microsynth (Microsynth Austria GmbH, Vienna, Austria) for Sanger sequencing in both directions. Consensus sequences were then aligned using ClustalX 2.1 [41].

### 2.6 Detection of WNV RNA

A multiplex RT-qPCR assay specific for WNV, DENV, and ZIKV was conducted using designated primers and probes for each virus. The following primers were used: for WNV lineage 1 and 2 detection, WNV-8-F (5’-CGCCTGTGTGAGCTGACAAA-3′), WNV-118-R (5’-GCCCTCCTGGTTTCTTAGACATC-3′), and WNV-67-P (5’-FAM-TGCGAGCTGTTTCTTAGCACGA-BHQ1–3′) as described by Kolodziejek et al. [42]; for DENV detection, the assay incorporated DENV-F (5’-GGAAGTAGAGCAATATGGTACATGTG-3′), DENV-R (5’-CCGGCTGTGTCATCAGCATAYAT-3′), and DENV-P (5’-HEX-TGTGCAGTCCTTCTCCTTCCACTCCACT-BHQ1–3′) following Pang et al. [43]; and ZIKV-specific amplification was performed using ZIKV-F (5’-CCGCTGCCCAACACAAG-3′), ZIKV-R (5’-CCACTAACGTTCTTTTGCAGACAT-3′), and ZIKV-P (5’-Cy5-AGCCTACCTTGACAAGCAATCAGACACTCAA-BHQ2–3′) according to Fournier et al. [44]. As positive controls, reagents for WNV, DENV serotypes 1–4, and ZIKV were sourced from Viasure (Certest Biotech, Zaragoza, Spain). Multiplex RT-qPCR was performed using the Luna Probe One-Step RT-qPCR Kit (New England BioLabs, Ipswich, MA, USA) on an LC480 II Light Cycler PCR System (Roche, Vienna, Austria). The cycling conditions were as follows: reverse transcription at 55 °C for 10 min (1 cycle), initial denaturation at 95 °C for 1 min (1 cycle), followed by 45 cycles of denaturation at 95 °C for 10 s and extension at 60 °C for 30 s, with fluorescence signal acquisition at each cycle.

WNV-positive samples were confirmed by conventional RT-PCR, using a specific primer pair designed to amplify the partial NS5 (viral polymerase gene) and the partial 3′-untranslated region (UTR) of WNV lineage 2 (forward primer: 5´-GARTGGATGACVACRGAAGACATGCT-3′ and reverse primer: 5´-GGGGTCTCCTCTAACCTCTAGTCCTT-3′) employing the QIAGEN OneStep RT-PCR Kit (Qiagen, Hilden, Germany) [45]. Amplicons (693 nt) were purified and sequenced by the Sanger method.

### 2.7 Phylogenetic analysis of WNV

The virus sequences were aligned to reference sequences taken from GenBank using MAFFT with the fast progressive method (FFT-NS-2). Reference sequences were selected to include whole genome sequences (trimmed to the 693 nt region) of WNV lineage 2 in Europe, using the strain isolated from a goshawk in Hungary, 2004, to root the tree as the first known sequence associated with the current WNV lineage 2 outbreak in central Europe [46]. The maximum likelihood tree was constructed using IQtree v 2.2.0.3 [47], which identified TIM2e + I as the best-fit substitution model with ModelFinder, and then inferred the consensus tree with 1000 ultrafast bootstrap iterations. The tree was visualized in ggtree (v3.10.1) and associated packages (tidytree v0.4.6 and treeio v1.26.0) in R statistical software (v4.3.3).

### 2.8 Haplotyping of *Aedes albopictus*

To visualize the geographic distribution of the *Ae. albopictus COI* lineages found in the present study, a DNA haplotype network was created following the approach of Bakran-Lebl et al. [48]. We downloaded all sequences of *Ae. albopictus* from NCBI GenBank, which covered a 624 bp section of the *COI* (nucleotide positions 70–693) and did not contain ambiguity characters and/or obvious sequencing errors (e.g. insertions, deletion, or stop codons). Country names and references were extracted from the GenBank files and information on the frequency of *COI* haplotypes was obtained from the respective populations. The sequences were aligned and sorted with MAFFT v.7.311 [49] using the default options, and the alignment was visually inspected with BioEdit v. 7.0.5.3 [50]. The entire data set comprised 2354 individual records from 44 peer-reviewed publications, 25 unpublished studies, and the present study. The partial 624 bp *COI* sequences clustered into 252 haplotypes, 144 of which were only detected once. To reduce the complexity of the network, we only included those 110 haplotypes (2210 individual records) which were found in at least two individuals (Supplementary Table S1). A Median Joining DNA haplotype network was calculated with NETWORK v.10.2.0.0 (Fluxus Technology Ltd., Cambridge, UK) and post-processed with the MP (maximum parsimony) option, which deletes non-MP links from the network. The haplotypes of the network were spatially arranged and provided with information on the geographic origin (United Nations Geoscheme) using NETWORK Publisher v.2.1.2.5 (Fluxus Technology Ltd), and visually edited with Adobe Illustrator CC v.2015.0.0 (Adobe Inc., San José, California, USA).

To show the number of records per country for the four *Ae. albopictus COI* haplotypes detected in the present study, pie charts were created using Microsoft Excel (Microsoft Office 365).

### 2.9 Statistical analysis and mapping of distribution

Data were analyzed with Microsoft Excel for Mac and R 3.6.2 [51]. For WNV, minimum infection rates (MIR) were calculated using the package “PooledInfRate“ available at https://github.com/CDCgov/PooledInfRate. Mapping of mosquito and pathogen distribution was performed with QGIS [52] using country and state/province data from www.naturalearth.com.

## 3 Results

### 3.1 Mosquito sampling, identification and barcode inventory

In total, 2691 mosquitoes (2592 females/99 males) were captured over 196 trap-nights. Mosquitoes were caught in 50 (89.3 %) of the 56 trapping sites. Morphological identification revealed 17 mosquito species from five genera: *Aedes*, *Anopheles*, *Coquillettidia*, *Culex*, and *Culiseta*. From all identified species *COI* barcode sequences were generated, uploaded to NCBI GenBank, and compared to reference sequences (Table 2).

**Table 2:** Identified mosquito species, number of sequenced barcodes, accession numbers, and percent sequence identity to reference sequences in GenBank.

Species	Total	Barcodes	Accession	% Identity (reference sequences)
*Ae. albopictus*	93	14	PV400675–PV400688	99.85–100 % (ON678140, ON678143)
*Ae. caspius*	10	2	PV400689–PV400690	100 % (MW961304, PP694675)
*Ae. cinereus*	4	3	PV400691–PV400693	99.54–99.85 % (MK403023, MK403147)
*Ae. geniculatus*	1	2	PV400694–PV400695	99.54 % (MW535828)
*Ae. japonicus*	2	2	PV400696–PV400697	100 % (MN103386, MK505599)
*Ae. pulcritarsis*	5	4	PV400698–PV400701	98.63–99.39 % (PP694673)
*Ae. sticticus*	3	3	PV400731–PV400733	99.85–100 % (KM258266, OK465163)
*Ae. vexans*	379	5	PV400702–PV400706	99.70–100 % (KY694975, MH348259)
*An. claviger* s.l.	109		–	–
*An. claviger* s.s.	14	4	PV400707–PV400710	99.39–99.70 % (PQ740514, MH643764)
*An. maculipennis* s.l.	489		–	–
*An. maculipennis* s.s.	2	2	PV400711–PV400712	99.70–99.85 % (OR200984, KM258134)
*An. messeae*^a^	2	2	PV400713–PV400714	99.54–99.70 % (PP941691, KU877008)
*An. plumbeus*	2	1	PV400715	100 % (ON126976, ON127158)
*Cq. richiardii*	29	2	PV400716–PV400717	99.85–100 % (OP204470, MK402670)
*Cx. modestus*	1	1	PV400718	99.39 % (MF537266)
*Cx. pipiens* s.l.	13	9	PV400719–PV400727	100 % (OM748626, MK714002)
*Cx. pipiens/torrentium*	916	3	–	–
*Cx. torrentium*	2	2	PV400728–PV400729	100 % (OM749546, OM749417)
*Cs. annulata*	177	1	PV400730	100 % (MK403465, OK465186)
*Aedes* spp.	289		–	–
*Anopheles* spp.	39		–	–
*Culiseta* spp.	2		–	–
Culicidae	62		–	–
Culicinae	43		–	–
**Total**	**2691**		**–**	**–**

a *Anopheles messeae/daciae* was further discriminated by ITS2.

The predominant species were *Culex pipiens*/*torrentium* (931/2592; 35.9 %), *Anopheles maculipennis* s.l. (492/2592; 19.0 %), and *Aedes vexans* (379/2592; 14.6 %) (Table 2).

### 3.2 Mosquito distribution

At mosquito-positive trapping sites, the species richness by location ranged from one (9 locations) to 11 (1 location) (Supplementary Fig. S1). Most locations were positive for *Culex pipiens* s.l. (44/56; 78.6 %) and *Anopheles maculipennis* s.l. (36/56; 64.3 %), whereas *Culiseta annulata* (27/56; 48.2 %), *Aedes vexans* (21/56; 37.5 %), and *Anopheles claviger* s.l. (19/56; 33.9 %) were also abundant at many trapping locations (Supplementary Fig. S1).

**Fig. 2:**
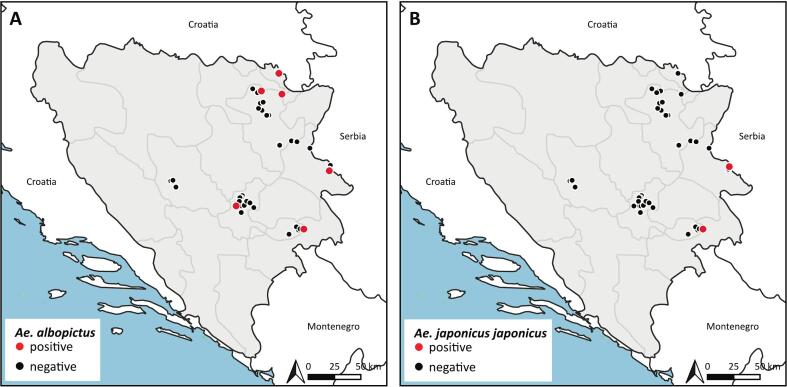
Positive trapping sites for (A) and *Ae. japonicus* (B) in this study.

*Aedes albopictus* was collected by human landing catches at seven sites and by light traps at two sites. The locations were in Orašje (three sites, northern BIH), one in Gradačac (northern BIH), two in Brčko (northern BIH), one in Bratunac (northeastern BIH), one in Goražde (eastern BIH), and one in Ilidža (central BIH) (Fig. 2A). In Brčko, *Ae. albopictus* was trapped in both years (2022 and 2023), while all other locations were only sampled in 2023. *Aedes japonicus* was found at two locations in 2022, namely one in Bratunac and one in Goražde (Fig. 2B).

### 3.3 *COI* haplotype network of *Ae. albopictus*

**Fig. 3:**
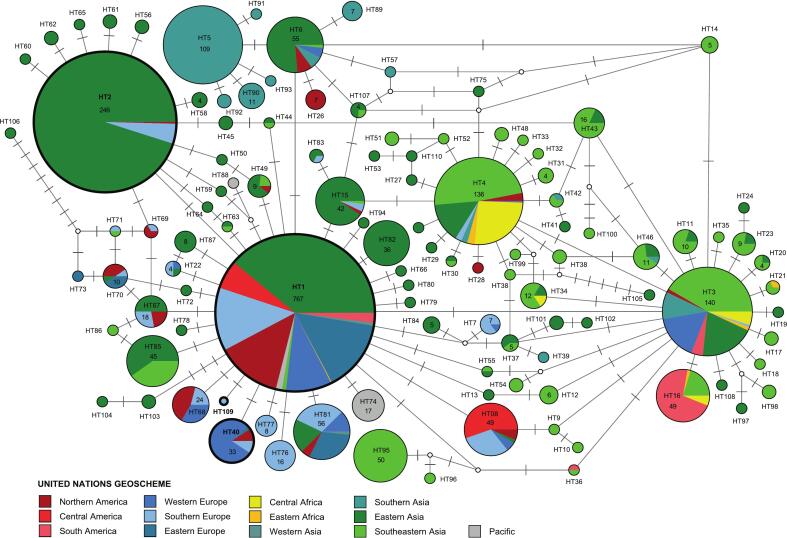
Median-Joining DNA haplotype network calculated with *COI* sequences (624 bp) of *Ae. albopictus*. The colored circles represent unique haplotypes. The haplotypes are numbered from HT1 to HT109. The size of the circles roughly corresponds to the number of individual records; the exact number is indicated for all haplotypes with four or more records. The colors correspond to the geographic division according to the United Nations Geoscheme. Small white circles are median vectors, which are hypothetical (ancestral or unsampled) sequences required to connect existing haplotypes with maximum parsimony. The bars on the branches indicate the number of substitutions separating two haplotypes. The haplotypes detected in the present study are encircled in bold.

**Fig. 4:**
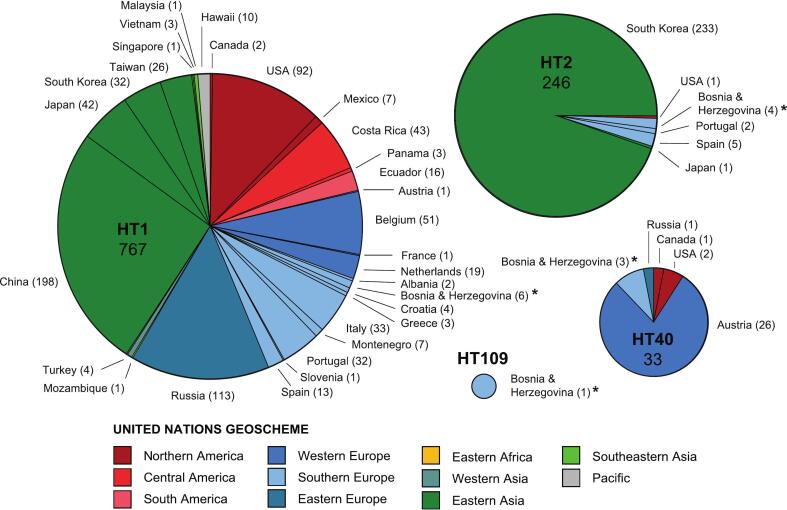
Number of records per country for the four *Ae. albopictus COI* lineages (624 bp) detected in the present study. The colors correspond to the geographic division according to the United Nations Geoscheme. The records of the present study are marked with asterisks.

**Fig. 5:**
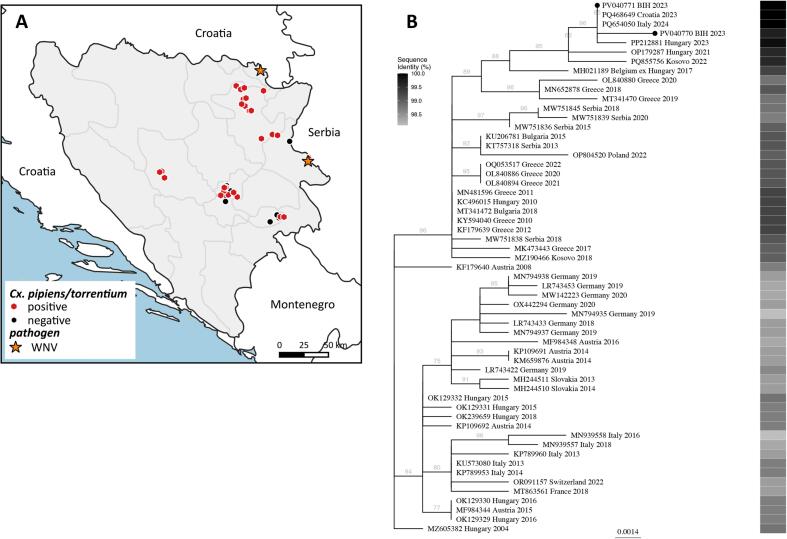
Trapping sites of *Cx. pipiens* s.l., and sites positive for WNV RNA (A). Maximum likelihood phylogenetic tree of West Nile virus lineage 2, including two sequences obtained from *Culex pipiens* s.l. captured in BIH in 2023 (black dots) (B). heatmap is shown beside the tree to indicate sequence identities (98.1 %–100.0 %, grey-to-black) of each strain to PV040771 over the 693 nt sequenced region, which includes 273 nt of the 3′ end of NS5 coding region and the partial 3′-untranslated region.

In the present study, four different *Ae. albopictus* haplotypes were recorded based on the 624 bp sequences analyzed in the *COI* haplotype network analysis. The network only included the 110 *COI* haplotypes, which were found at least two times. The 144 haplotypes detected only once previously were discarded from the analysis, except for the new lineage (haplotype HT109) found in the present study. The sequences of all *Ae. albopictus COI* haplotypes and related information are listed in Supporting Information Table S1. The four haplotypes are closely related, whereby HT2, HT40, and HT109 each differ by 1 bp from HT1 (Fig. 3). The haplotypes designated as HT1 and HT2 represent the most frequently recorded *Ae. albopictus* haplotypes worldwide. Haplotype HT1 (767 records) has been found in Northern America (101), Central America (46), Southern America (16), Western Europe (72), Southern Europe (101), Eastern Europe (113), Eastern Africa (1), Western Asia (4), Southern Asia (1), Eastern Asia (298), Southeastern Asia (5), and Hawaii (10). In Europe, HT1 has been detected in 16 countries, including Albania, Croatia, Greece, Montenegro, Slovenia, and BIH (6 individuals; present study, Fig. 5) in the Balkan area. Haplotype HT2 (246 records) has been found in Eastern Asia (234), Southern Europe (11), and Northern America (1). In Europe, HT2 has only been recorded in Spain, Portugal, and BIH (4 individuals; present study, Fig. 4). The third haplotype, HT40 (33 records), has been found in Western Europe (26), Southern Europe (3), Eastern Europe (1), and Northern America (3). In Europe, HT40 has been previously recorded in Austria (26) and Russia (1); the three specimens from BIH represent the first record of this haplotype in Southern Europe. The fourth haplotype, HT109, has only been found in a single specimen in the present study.

### 3.4 RNA-based pathogen detection

Of 931 *Culex pipiens*/*torrentium* specimens (179 pools) screened, two (2/179; 1.1 %, MIR = 2.2, 95 % CI: 0.4–7.2) pools of 16 and 20 female specimens, respectively, were positive for WNV lineage 2 RNA. Positive pools originated from locations in Bratunac (PV040770) and Orasje (PV040771) (Fig. 5A). The sequences differed from each other at two sites along the 693 nt sequenced region (99.7 % sequence identity): PV040770 had C10266T and T10463C compared to PV040771 and all other reference strains from Europe. The C10266T substitution is a synonymous mutation at position 861 in the mature NS5 (viral polymerase) peptide, whereas the T10463C is in the 3′ untranslated region. Both sequences formed a strongly-supported clade (95 % bootstrap support) with 100 % sequence identity to reference sequences identified in Italy 2024 (e.g., PQ654050) and Croatia 2023 (PQ468649), as well as high sequence identity (≥99.6 %) to a sequence from Kosovo 2022 (PQ855756) and sequences from Hungary 2021–2023 (PP212001 and OP179287) (Fig. 5B).

### 3.5 DNA-based pathogen screening

**Fig. 6:**
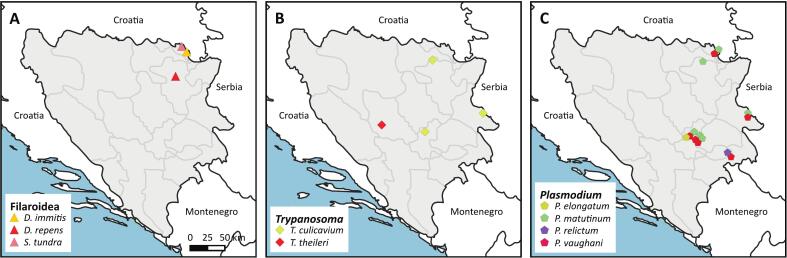
Trapping sites of mosquito pools positive for *D. immitis*, *D. repens*, and *S. tundra* (A), *Trypanosoma* spp. (B), and *Plasmodium* spp. (C).

Overall, two pools of *An. maculipennis* s.l. from two locations in Orašje (Fig. 6A) were positive for *D. immitis* (PV400735); the *COI* sequences of these pools were identical to those of *D. immitis* isolated from *Aedes* mosquitoes in Hungary (MH541832, KM452921) and the USA (PP869405–06), cats in France (PQ560497–98), golden jackals in Iran (KT351849–52), and dogs in Iran, Bangladesh, Hong Kong, and South Korea.

One *Ae. vexans* pool from a location in Srebrenik (Fig. 6A) was positive for *D. repens* (PV400734); the *COI* sequence was identical to that of a dog in the Czech Republic (MW675691). Another *Ae. vexans* pool from Orašje (Fig. 6A) was positive for *Setaria tundra* (PV400736); the sequence was identical to that of *S. tundra* from Germany (KF692104), Austria (MF695096), and Poland (MK360915).

Three *Cx. pipiens*/*torrentium* pools from locations in Tuzla and Orašje (Fig. 6A) were positive for *Trypanosoma culicavium* (PV610735); an identical 18S sequence was isolated from a barn swallow in the Czech Republic (OM509727). One *An. maculipennis* s.l. pool from a location in Bugojno (Fig. 6B) was positive for *T. theileri* (PV610736); the sequence shared 99.89 % similarity with a sequence isolated from a cow in Scotland (AJ009163). Additionally, monoxenous trypanosomatid species were found. Most of them belonged to the genus *Crithidia*, namely *Crithidia brevicula* (PV610732), *Crithidia dedva* (PV610733), and *Crithidia dobrovolskii* (PV610734). *Crithidia brevicula* was found in one *Cx. pipiens/torrentium* pool, whereas *C. dedva* and *C. dobrovolskii* were found throughout the country in nine and fourteen different locations, respectively. Another trypanosomatid, *Blastrocrithidia culicis* (PV610731), was detected in a pool of *Ae. vexans*.

Avian *Plasmodium* parasites were detected in eighteen *Cx. pipiens*/*torrentium* pools throughout the country, at locations in Orašje, Bratunac, Sarajevo, and Goražde (Fig. 6C). The most common *Cytb* lineages were *P. matutinum* pLINN1 (PV611340) and *P. vaughani* pSYAT05 (PV611338), while *P. elongatum* pGRW06 (PV611339) was found in one pool. One *Ae. albopictus* pool from Goražde was positive for *Plasmodium* sp. pCOLL1 (PV611341).

### 3.6 Blood meal analysis

Blood meals were analyzed from 62 engorged individual mosquito specimens from nine species encountered in the light traps. Of these, 42 blood meals were identified comprising nine host species, mostly cow (*Bos taurus,* 12 blood meals) and sheep (*Ovis aries*, 11 blood meals) (Table 3). Most blood meals and host species were identified from *Ae. vexans* (14), comprising DNA of seven hosts, namely mammalian hosts such as dog (*Canis lupus familiaris*), cow, sheep, wild boar (*Sus scrofa*), and humans as well as farmed wild turkey (*Meleagris gallopavo*) and chicken (*Gallus gallus*) (Table 3). *Anopheles maculipennis* s.l. fed on four hosts including cow, goat (*Capra hircus*), sheep, and wild boar. One blood-fed *An. claviger* s.l. specimen was positive for European rabbit (*Oryctolagus cuniculus*), and hosts of *Cx. pipiens* s.l. were identified as cow (1) and chicken (1). For *Ae. albopictus*, one human blood meal was identified (Table 3). We note that the sequenced region cannot discriminate between wild boar and domestic pigs nor between wolf and domestic dog.

**Table 3:** Identified hosts of various mosquito species identified by blood meal analysis.

Mosquito species	Host								
	Human	Dog	Cow	Sheep	Goat	Pig	Rabbit	Turkey	Chicken
*Ae. albopictus*	1	0	0	0	0	0	0	0	0
*Ae. pulcritarsis*	0	0	1	0	0	0	0	0	0
*Ae. sticticus*	0	0	0	1	0	0	0	0	0
*Ae. vexans*	3	3	2	2	0	1	0	1	2
*An. claviger s.l.*	0	0	0	3	0	0	1	0	0
*An. daciae/messeae*	0	0	0	0	0	1	0	0	0
*An. maculipennis s.l.*	0	0	6	3	2	1	0	0	0
*Cq. richiardii*	0	0	0	1	0	0	0	0	0
*Cx. pipiens/torrentium*	0	0	1	0	0	0	0	0	1
*Cs. annulata*	0	1	2	1	1	0	0	0	0
total	**4**	**4**	**12**	**11**	**3**	**3**	**1**	**1**	**3**

## 4 Discussion

The present study provides a comprehensive overview of the underreported mosquito fauna in three of five geographical regions of BIH, highlighting the presence and distribution of mosquito species incriminated in the transmission of important pathogens such as West Nile virus and *Dirofilaria* spp. which includes the non-native *Aedes albopictus* and *Ae. japonicus* *japonicus* as well as endemic species. To our knowledge, this is the first report confirming the presence of West Nile virus RNA, *Dirofilaria* spp., and *Setaria* spp. in mosquitoes from BIH. In addition, *Trypanosoma* and *Plasmodium* parasites were detected in some of the pooled samples.

Entomological surveys to assess the mosquito fauna in BIH are rare, thus, the true species diversity of the country is likely underreported. Given the shared climatic conditions and biogeographical features throughout the Balkans, a significant overlap in the mosquito fauna between BIH and its neighboring countries can be expected, although the species composition may be influenced by regional biological factors. In our study, we identified 17 mosquito species of six genera, with *Cx. pipiens*/*torrentium* being the most frequently recorded mosquitoes, followed by *An. maculipennis* s.l., and *Cs. annulata*. The high abundance of *Culex* species is important given their role as vectors of WNV, which is frequently reported in Balkan countries. A three-year survey in the Vojvodina region of the Republic of Serbia, bordering our study region in northeastern BIH, reported 20 mosquito species [7], and 13 species of six genera were trapped in a baseline study in the Republic of Kosovo [53]. In both surveys, *Cx. pipiens* s.l. was the predominant species collected in light traps, which aligns well with our results, reporting *Cx. pipiens*/*torrentium* from various habitats, highlighting its adaptability to urban and peri-urban environments. Similarly, studies conducted in the Istrian peninsula [54] and in Lika, central Croatia [55], have consistently reported a high prevalence of mosquitoes belonging to the *Cx. pipiens* complex.

Contrary to the highly abundant *Culex* species, *Coquillettidia richiardii* was detected in low numbers, consistent with findings from other European studies where the species is typically reported in wetland habitats with abundant emergent vegetation [56]. This species has been identified as a potential bridge vector for arboviruses in parts of Europe [57,58], although its epidemiological relevance in the Balkans remains poorly understood. Other species, such as *Ae. cinereus* and *Ae. geniculatus* were also detected in low numbers, which could be related to their association with localized breeding sites [59]. The absence or lower abundance of certain species common in adjacent countries may be attributed to factors such as local climatic conditions, host-feeding preferences, or historical dispersal patterns, but also sampling effort might display a bias, particularly light trapping performed in this study adds a bias that should be taken in consideration. If future sampling initiatives are targeted toward forested areas and more diverse habitats, it may help clarify the distribution and potential vectorial capacity of these species in BIH [60].

We report the presence of *Ae. albopictus* in northern and eastern BIH for the first time, and the first records of *Ae. japonicus japonicus* in eastern BIH. *Aedes albopictus* is a mosquito species of global concern and has demonstrated a complex introduction history in Europe, with multiple independent introduction events shaping its current genetic diversity and distribution [61]. While our haplotype analysis is solely based on COI sequences, we hypothesize that *Ae. albopictus* populations in BIH might be primarily derived from secondary introductions within Europe rather than direct arrivals from its native range in Southeast Asia. This aligns with findings from Bakran-Lebl et al. [48], who identified shared haplotypes, namely HT01 and HT40, between Austria and BIH, suggesting dispersal through trade and vehicular transport rather than primary introductions from Asia. However, the detection of a previously unrecorded haplotype (HT109) in BIH highlights the possibility of sporadic direct introductions from under-sampled regions in Asia, emphasizing the ongoing risk of novel genetic variants being introduced into European populations, which should be supported by deeper population genetic studies using multiple loci, microsatellites or even mitogenomes [62]. Yet, the evolutionary stability of the *COI* gene further supports the assumption that most haplotypes already existed when *Ae. albopictus* was initially introduced to Europe, and that continued dispersal is largely driven by intra-European movements, particularly through commercial exchanges and human activity [63]. Similar introduction pathways have been observed across the Balkans, where *Ae. albopictus* has rapidly expanded its range, facilitated by climatic conditions conducive to its establishment and artificial breeding sites in urban environments [3,64,65].

In contrast, *Ae. japonicus*, also detected in our study, might be in an earlier phase of its invasion trajectory, with more localized populations compared to the widespread presence of *Ae*. *albopictus*, however intensified entomological surveys are urgently needed to assess the actual current distribution. The growing presence of both species across the region underscores the importance of continued genetic monitoring and vector control efforts to mitigate potential public health risks associated with these invasive mosquitoes [66,67].

Contrary to findings from other European countries, *Ae. koreicus*, an introduced species known to be spreading in parts of South Europe [68,69], was not detected in this study. Despite its confirmation in Slovenia and Italy [70], its absence in our sampling could be due to the sampling methods, environmental factors, or limited establishment in BIH.

The bordering countries Serbia and Croatia have previously reported WNV detection in humans, horses, birds, and mosquito vectors [[71], [72], [73], [74], [75]] as well as extensive surveillance activity [72,76]. However, to our knowledge, only two patients were diagnosed with West Nile neuroinvasive disease in BIH in 2013 (by detection of WNV-reactive IgM in serum) [18]. Herein, we present the first evidence of the virus in BIH by detection of viral nucleic acids in *Culex* mosquitoes. We confirmed the WNV-positive mosquito pools using RT-qPCR as well as conventional RT-PCR followed by sequencing, as is routinely performed in surveillance studies. Therefore, although we did not isolate the virus, we can be reasonably sure that we identified the virus in questing female mosquitoes in BIH. Moreover, the virus sequence was similar (>98 % identity) to the Central European/Hungarian clade of WNV lineage 2 that has been continuously circulating in Europe since its introduction in at least 2004 [46]. Moreover, both sequences generated in our study showed that the virus had high sequence identity (>98.8 %) and formed a strongly supported clade (>95 % bootstrap support) to other WNV lineage 2 sequences identified from mosquitoes, birds, humans, and horses in southeastern Europe over the last 10 years displayed in Fig. 4. Therefore, it is unlikely to represent a recent introduction or chance finding, and efforts to further characterize WNV in BIH should be undertaken.

Previous studies have documented the presence of *Dirofilaria* in mosquito vectors across south-eastern Europe, including Serbia [29]. Human dirofilariasis has been reported increasingly in patients in the last few years in neighboring countries [[77], [78], [79]], suggesting that the Balkans serve as an endemic region for these parasites. In our study, *Dirofilaria* was detected in *Ae. vexans* and *An. maculipennis* s.l., both of which have been implicated as vectors in Europe. Notably, *Ae. vexans* has been identified as a carrier of *D. repens* in the Czech Republic and Slovakia [80,81]. Similarly, *An. maculipennis* s.l. has been found to harbor both *D. repens* and *D. immitis* in Austria and Moldova [82,83]. In addition to *Dirofilaria*, molecular screening also revealed the presence of *Setaria tundra*, another filarioid parasite with veterinary significance. Similar to our finding, *S. tundra* was recently detected in *Ae. vexans* in Serbia by Šiljegović et al. [29], adding to the role of mosquitoes as vectors of filarial nematodes beyond the commonly studied *Dirofilaria* species. While *S. tundra* is primarily associated with cervids [84], its detection in mosquitoes highlights the need for further research into its potential spillover to domestic animals and its broader ecological impact. The widespread distribution of competent mosquito species raises concerns about the ongoing transmission cycle of *Dirofilaria* in BIH, particularly in peri-urban and rural areas where suitable reservoir hosts, such as dogs and wild canids, are abundant. Further research is essential to determine the prevalence and potential impact of both *Dirofilaria* spp. and *Setaria* spp. infections in local wildlife and livestock populations, to ensure a more comprehensive assessment of their transmission dynamics and veterinary significance.

Various bloodsucking insects and ticks have been reported to harbor trypanosomes, and mosquitoes are not considered important vectors [85]. Although only a few mosquito pools were positive for trypanosomatids in the present study, the issue requires further discussion. While *Culex* mosquitoes are the main vectors of *T. culicavium*, a parasite of birds [86], species of the *T. theileri* group have been detected in various dipteran groups infecting mammalian hosts such as cervids or bovids [87]. Our findings of *T. culicavium* in *Culex* spp. is in line with the literature. Additionally, one *An. maculipennis* s.l. pool was positive for *T. theileri*, which has been previously detected in five different Culicidae genera including *Anopheles*. The veterinary relevance of these trypanosomatids is yet unclear, infections by *T. theileri* are reported to be mostly cryptic. Reported pathologies such as fever, anorexia, and anemia were assumed to be associated with coinfections or stress of infected bovids [[88], [89], [90]].

While avian *Plasmodium* parasites do not pose a direct threat to human health, they can significantly impact wild and domestic bird populations [91]. Particularly, an introduction of avian malaria parasites to areas with immunologically naïve individuals might promote an excessive population decline of native avian populations [92]. We detected four different species, most commonly *P. matutinum* pLINN1 and *P. vaughani* pSYAT05 in *Culex pipiens*/*torrentium* pools. Both of the latter parasites are particularly common in Eurasian blackbirds but can severely impact the health of zoo-housed non-native birds such as African penguins (*Spheniscus demersus*) and lovebirds (*Agapornis roseicollis*), thus urging for control and prevention strategies in institutions like zoos or wildlife shelters [93,94]. Interestingly, we detected the lineage *Plasmodium* sp. pCOLL1 in one *Ae. albopictus* pool. While the competence of *Ae. albopictus* to transmit avian *Plasmodium* parasites is unclear, transmission has been proven for some species under laboratory conditions [95]. However, the anthropogenic introduction of *Ae. albopictus* to new areas could facilitate local transmission of these pathogens among many others (e.g. Dengue virus) in invaded areas, if vector competence is given, and thus requires surveillance.

## 5 Conclusion

The findings of this study highlight an urgent need for One Health-based approaches, assessing connectivity between mosquito species, animal and human hosts as well as environmental factors promoting establishment of vector-borne transmission cycles. The detection of filarial nematodes, protozoan parasites, and arboviruses in mosquito populations indicates that BIH, like other parts of Europe, is at risk of emerging vector-borne diseases [56,96]. The presence of WNV in *Culex* mosquitoes further emphasizes the role of BIH in the virus' epidemiology within southeastern Europe, particularly given the widespread distribution of *Cx. pipiens*, a key vector in previous WNV outbreaks across the Balkans [97]. The established presence of two competent vectors of various arboviruses and filarial nematodes – *Ae. albopictus* and *Ae. japonicus* – in BIH, poses an increasing risk of vector-borne disease transmission [98]. Additionally, the detection of *Dirofilaria* species reinforces the growing concern about filarioid infections in Europe, where climate change and the expansion of invasive mosquito species may facilitate their spread [99]. This baseline data serves as a crucial resource for future surveillance efforts aimed at developing strategies to mitigate the risks posed by vector-borne pathogens.

## Ethical approval

Not applicable.

## Funding

The study has been funded by the Austrian defence research program FORTE of the Federal Ministry of Finance (BMF) [grant number 886318]. The funders had no role in the study design, data collection and analysis, decision to publish, or preparation of the manuscript.

## Informed consent statement

Verbal consent was obtained from all homeowners to trap at their properties.

## CRediT authorship contribution statement

**Ina Hoxha:** Writing – review & editing, Writing – original draft, Methodology, Formal analysis, Conceptualization. **Jovana Dervović:** Writing – review & editing, Methodology. **Maria Sophia Unterköfler:** Writing – review & editing, Methodology. **Lisa Schlamadinger:** Writing – review & editing, Methodology. **Tanto Situmorang:** Writing – review & editing, Methodology. **Hans-Peter Fuehrer:** Writing – review & editing, Methodology, Formal analysis. **Adelheid G. Obwaller:** Writing – review & editing, Supervision, Project administration. **Karin Sekulin:** Writing – review & editing, Methodology. **Jeremy V. Camp:** Writing – review & editing, Writing – original draft, Methodology, Formal analysis. **Josef Harl:** Writing – review & editing, Methodology. **Julia Walochnik:** Writing – review & editing, Supervision, Project administration. **Amer Alić:** Writing – review & editing, Writing – original draft, Supervision, Project administration, Methodology, Conceptualization. **Edwin Kniha:** Writing – review & editing, Writing – original draft, Supervision, Project administration, Methodology, Conceptualization.

## Declaration of competing interest

The authors declare that they have no known competing financial interests or personal relationships that could have appeared to influence the work reported in this paper.

## Data Availability

All data are available in the manuscript and the supplementary files.

## Appendix A Supplementary data

Supplementary material Image 1
