# Supplementary figures and images for: A cross section through mosquitoes of Bosnia and Herzegovina: Barcodes, blood meals and pathogens
Source: One Health. 2025 Oct 15;21:101246. doi: 10.1016/j.onehlt.2025.101246 (PMC12555878; doi:10.1016/j.onehlt.2025.101246)

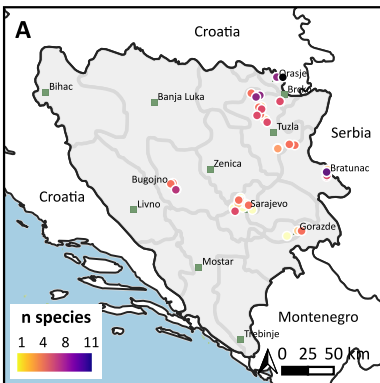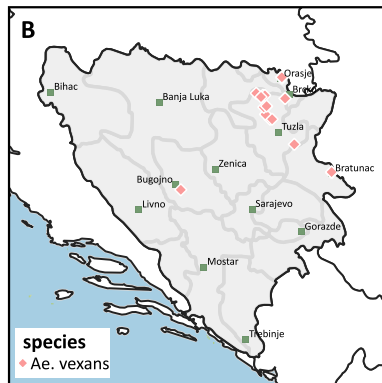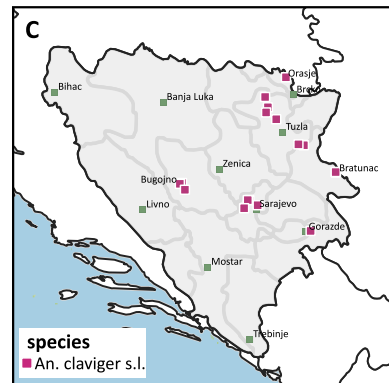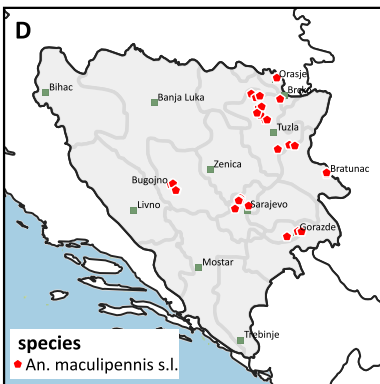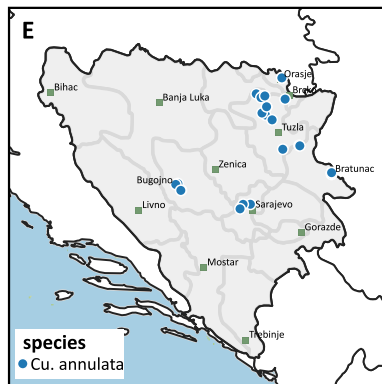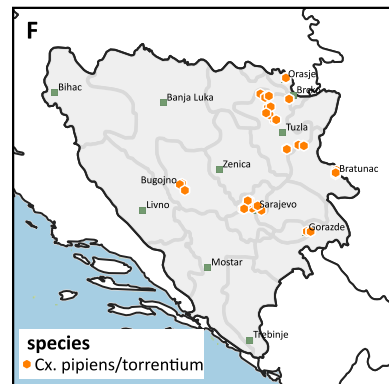

Supplement: Supplementary file 1 — Supplementary material [file mmc1.pdf]
